# Late antenatal care initiation and its contributors among pregnant women at selected public health institutions in Southwest Ethiopia

**DOI:** 10.11604/pamj.2021.39.264.22909

**Published:** 2021-08-24

**Authors:** Melkamsew Tesfaye, Yadeta Dessie, Melake Demena, Tewodros Yosef

**Affiliations:** 1 Department of Nutrition and Reproductive Health, School of Public Health, College of Medicine and Health Sciences, Mizan-Tepi University, Mizan-Aman, Ethiopia,; 2 School of Public Health, College of Health and Medical Sciences, Haramaya University Harar, Ethiopia,; 3 School of Post Graduate, College of Health and Medical Sciences, Haramaya University, Harar, Ethiopia,; 4 Department of Epidemiology and Biostatistics, School of Public Health, College of Medicine and Health Sciences, Mizan-Tepi University, Mizan-Aman, Ethiopia

**Keywords:** Antenatal care, late initiation, pregnant, Ethiopia

## Abstract

**Introduction:**

early commencement of antenatal care by pregnant women as well as regular visits has the potential to affect maternal and fetal outcomes positively. Even with antenatal care, the intervention requires fewer resources; however, most pregnant women in sub-Saharan Africa have begun late for antenatal care services. This study aimed to assess the magnitude and contributors of late antenatal care initiation among pregnant women at selected public health institutions of the Bench-Sheko Zone in southwest Ethiopia.

**Methods:**

a cross-sectional study was conducted among 509 pregnant women attending the Antenatal Care (ANC) service at selected public health institutions. The data were collected using a structured and pre-tested questionnaire. The data were entered using Epi-data version 3.1 and analyzed using SPSS version 22. A binary logistic regression analysis was computed to determine the association using crude and adjusted odds ratios at 95% confidence intervals. Independent variables with a p-value of less than 0.05 in the multivariable logistic regression model were considered significant.

**Results:**

of the 509 respondents interviewed, 337 (66%) reported late antenatal care initiation. The factors associated with late antenatal care initiation were mothers aged 25 years and above (AOR = 1.59, 95% CI [1.02, 2.48]), attended below secondary school (AOR =2.33, 95% CI [1.05, 5.19]), unplanned pregnancy (AOR=2.25, 95%CI [1.34, 3.77]), pregnancy recognition by missing period (AOR=0.61, 95%CI [0.39, 0.93]), perceived right time of ANC after 4 months (AOR=2.29, 95% CI [1.36, 3.85]), and did not get advice to have ANC (AOR=1.64, 95% CI [1.10, 2.45]).

**Conclusion:**

the majority of pregnant women initiate their first antenatal care lately. We can conclude that late antenatal care initiation is a major problem in the study area. Therefore, providing continuous health education on the importance of initiating antenatal care visits early to prevent unwanted pregnancy outcomes is an important segment of intervention that can be done through health extension workers.

## Introduction

Maternal mortality is the leading cause of death among women aged 15-49 years old, with an estimated 303,000 maternal deaths occurring in 2015 alone. Almost all (99%) of maternal deaths occur in developing countries and sub-Saharan Africa accounted for 66% of maternal deaths [1]. In Ethiopia, maternal mortality and morbidity levels are the highest in the world. In 2016 alone, the maternal mortality ratio (MMR) was 412 per 100,000 live births [2]. Women die as a result of complications during and following pregnancy and childbirth. Most of these complications develop during pregnancy and most are preventable or treatable. Other complications may exist before pregnancy but are worsened during pregnancy, especially if not managed as part of the woman´s care [3]. Thus, a pregnant woman should receive special care and attention from the family, community, and health care system [4]. ANC is a complex of interventions that a pregnant woman receives from organized health care services intended to assure every pregnancy culminates in the delivery of a healthy child without impairing the health of the mother [5].

Antenatal care from skilled providers at health facilities is a priority in ensuring and enriching materno fetal health. Early detection of problems in pregnancy is used for more timely referrals for women in high-risk categories or with complications [6]. The timing of the first visit is around or preferably before 16 weeks of gestational age [7]. Early commencement of antenatal care by pregnant women as well as regular visits has the potential to affect maternal and fetal outcomes positively [8, 9]. Late initiation of ANC may lead to undetected or late detection of fetal and maternal health problems [10]. Despite antenatal care service is a less resource-intensive intervention provided throughout the pregnancy period to reduce maternal mortality [11], however, most pregnant women in sub-Saharan Africa have begun ANC service utilization lately [12, 13]. According to the Ethiopia Demographic and Health Survey (EDHS) 2016, only 20% of women made their first ANC visit before the fourth month of pregnancy, and the remaining 80% of pregnant women are lately initiated their first antenatal care [2]. The magnitude of late antenatal care initiation ranges from 42% to 81.5% in Ethiopia [4, 9, 14-19] and 80% in Nigeria [20].

The factors associated with first antenatal care initiation are diverse and include education, age, place of residence, employment status, parity, intention to get pregnant, means of pregnancy recognition, economic status, perceived right time to get ANC, previous utilization of ANC, poor knowledge about ANC, and travel expenses [13-15, 18, 20-23]. Early initiation of ANC has the benefit of early detection and treatment of complications during pregnancy, however, antenatal care utilization is generally low, and delayed initiation of care is very common in Ethiopia [22, 24]. Despite studies done regarding late ANC initiation [13-15, 18, 24], almost all were done in urban communities. But this study mainly included more of the rural communities. That´s why we have studied these populations with the expectation of getting a different result. Since the cultural and socioeconomic characteristics of the study population were different from populations those of other studies. Therefore, this study aimed to assess the magnitude and contributors of late antenatal care initiation among pregnant women at selected public health institutions of the Bench-Sheko Zone in southwest Ethiopia.

## Methods

**Study design, setting, and period**: a facility-based cross-sectional study was conducted at selected public health institutions in the Bench-Sheko Zone from February 1 to March 2, 2018. Bench-Sheko Zone is found in the southern nation´s nationalities and people´s regional state at roughly 585 kilometers southwest of Addis Ababa; the capital city of Ethiopia, and 84>9 kilometers from the regional capital Hawassa. The Bench-Sheko Zone is administratively divided into six woredas (districts) and two town administrations. The total population in the study area was projected to be 847,168 (417,751 males and 429,417 females) in 2017. The zone has eleven health centers and one teaching hospital owned by the government.

**Populations**: the source of the population was all pregnant mothers, who were attending ANC visits at Bench-Sheko Zone selected public health facilities. The study population was all pregnant women who attended their first ANC visit at selected public health care facilities during the data collection period. All pregnant women who will come to their first ANC visit and whose gestational age is identified either by last menstrual period or ultrasound in the selected health facility during the data collection time were included in the study. Pregnant women who have not known gestational age either by their last menstrual period or ultrasound and mentally incapable women were excluded.

**Sample size determination and sampling method**: the required sample was determined using a single population proportion formula with an input of expected proportion of late initiation of ANC in Halaba, Ethiopia to be 73% [4], a margin of error (4%), 95% confidence interval, 10% for non-response compensation. The sample size computed was 521. A simple random sampling was followed to select health institutions. The study area has 1 teaching hospital, 1urban, and 10 rural health centers. Six health centers (1 urban and 5 rural) randomly and 1 teaching hospital purposively were selected. The number of study participants was allocated proportionally based on the previous year's ANC client flow report. Then, the eligible pregnant women who came for their first ANC visit in the selected health centers were enrolled continuously until the required sample size was achieved.

**Data collection instrument and procedures**: the instrument of data collection was an interviewer-administered, pre-tested structured questionnaire. The questionnaire was developed based on the EDHS [2] data collection tool and other relevant literature [9, 24]. The questionnaires contained the socio-demographic, obstetric, and current pregnancy-related characteristics, health care facility-related factors, perception of ANC service. The household wealth index was assessed using principal component analysis. The questionnaires were initially prepared in English and then translated into Amharic and back-translated into English to check the consistency by an independent translator to check its consistency. To increase the quality of the data, the questionnaire was pre-tested in similar setups before the actual data collection was commenced. The training was given to data collectors and supervisors concerning the objective and process of data collection and discusses the presence of an ambiguous question in the questionnaire and if there, was clarified.

**Study variables and measurements**: the dependent variable was the late antenatal care initiation. The independent variables were predisposing factors (maternal age, gravidity, educational status, marital status, previous use of ANC services, residence, pregnancy recognition), enabling factors (household wealth index, waiting time for ANC service, occupation, means, and service cost), and need factors (pregnancy intention, mode of the previous delivery, perceived right time to start ANC, get advised on ANC, previous pregnancy complication).

**Late initiation**: initiation of first ANC visit after 16^th^ weeks of gestation, otherwise early initiation. Knowledge: pregnant women´s awareness about ANC visits and the danger signs of pregnancy. Good knowledge: pregnant mothers with scores greater than or equal to the mean value of the knowledge measuring questions, otherwise poor knowledge.

**Data processing and analysis**: the collected data were entered into the Epi-data version 3.1 to prevent data entry error, missing data, and avoiding inappropriate values by fixing the range of values coded to the software and analyzed using SPSS version 22. Descriptive statistics of the independent variables were presented by frequency and percentage using tables and pie charts. Binary logistic regression analysis was used to look for the association between outcome and independent variables. Independent variables with a p-value < 0.25 in the bivariate logistic regression were included in the multivariable logistic regression to control for potential confounding factors and to identify the most important determinate variables. Finally, variables in the multivariable logistic regression with a p-value < 0.05 were considered as significantly associated with the outcome variable. Multi-collinearity between independent variables in the model was checked, and the variance inflation factor (VIF) was found acceptable (less than 2). The Hosmer-Lemeshow goodness-of-fit test indicated (P=0.972) that the model was good enough to fit the data well.

**Ethical consideration**: ethical approval was obtained from the Haramaya University-Institutional Review Board (HU-IRB) on 04/01/2018 with the reference number HUIRB/083/2018. A permission letter was obtained from the Bench-Sheko Zone Health Bureau. All study participants were informed about the purpose of the study, their right to deny participation, anonymity, and confidentiality of the information. Written informed consent was also obtained before participation in the study.

## Results

**Sociodemographic characteristics**: of the 521, 509 pregnant mothers were interviewed to make a response rate of 97.7%. Three hundred nine (60.7%) were in the age group of 25 years and above, with a median age of 25.8 years. The majority (51.9%) of the participants were protestant religious followers. The majorities (94.1%) of the participants were married and almost half (49.5%) of the study participants had never attended formal education (Table 1).

**Table 1:** socio-demographic characteristics of pregnant women attending ANC services at selected public health institutions in southwest Ethiopia

Variables	Categories	Frequency	Percent
**Age**	< 25 years	200	39.3
	≥ 25 years	309	60.7
**Marital status**	Married	479	94.1
	Unmarried	30	5.9
**Religion**	Orthodox	166	32.6
	Protestant	264	51.9
	Muslim	79	15.5
**Ethnicity**	Bench	211	41.5
	Sheko	62	12.2
	Amhara	116	22.8
	Keffa	120	23.6
**Maternal Education**	No formal education	252	49.5
	Primary (1-8)	122	24
	Secondary(9-12)	62	12.2
	College and University	73	14.3
**Husband Education**	No formal education	174	34.2
	Primary (1-8)	131	25.7
	Secondary(9-12)	82	16.1
	Above Secondary	122	24
**Residence**	Urban	72	14.1
	Rural	437	85.9

**Obstetric and current pregnancy-related data**: three hundred forty (66.8%) had a history of previous pregnancy, and 79.4 % of them had previously used ANC. Almost two-thirds (64.8%) of the participants recognized their pregnancy via a urine test. A majority (74.7%) of the participants had planned pregnancies and 9.4% of the study subjects had a history of abortion. The majority (96.3%) of the participants gave birth through assisted spontaneous vaginal delivery during their previous delivery (Table 2).

**Table 2:** obstetric and current pregnancy-related data of pregnant women attending ANC services at selected public health institutions in southwest Ethiopia

Variables	Categories	Frequency	Percent
Gravidity (n=509)	Primigravida	169	33.2
	Multigravida	340	66.8
Pervious ANC utilization (n=340)	Yes	270	79.4
	No	70	20.6
Pregnancy (n=509)	Planned	380	74.7
	Unplanned	129	25.3
History of Abortion (n=340)	Yes	32	9.4
	No	308	90.6
Mode of previous delivery (n=340)	SVD	321	96.3
	CS/Instrumental	19	5.6
Previous Pregnancy Complication (n=340)	Yes	44	12.9
	No	296	87.1
History of child death (n=340)	Yes	53	15.6
	No	287	84.4
Means of Pregnancy recognition(n=509)	Missing period	340	64.8
	Urine test	169	35.2

SVD: spontaneous vaginal delivery, CS: cesarean section, Instrumental: forceps

**Knowledge and perception of ANC service**: three hundred seventy (72.7%) participants rated that ANC visits as important for the mother as well as the fetus. Of these, 72 % of them had an awareness of the recommended time of ANC initiation, and 37.5% of pregnant women had known obstetric danger signs. Two-hundred ninety-three (57.6%) perceived four or more ANC visits required throughout the whole pregnancy time. The mean knowledge of the respondents was 4.4, and 256 (50.3%) of them had good knowledge about antenatal care visit timing (Table 3).

**Table 3:** knowledge and perception of ANC service utilization of pregnant women attending ANC services at selected public health institutions in southwest Ethiopia

Variables	Categories	Frequency	Percent
**For whom ANC is important**	Fetus	30	5.9
	Mother	109	21.4
	Both	370	72.7
**The perceived right time to start ANC (n=509)**	Within 4 months	367	72.1
	After 4 months	142	27.9
**Perception on number of ANC visits during pregnancy (n=509)**	1	8	1.6
	2	22	4.3
	3	119	23.4
	≥4	293	57.6
	I don´t know	67	13.2
**Awareness of obstetric danger signs (n=509)**	Yes	191	37.5
	No	318	62.5
**Did ANC used for birth preparedness (n=509)**	Yes	480	5.7
	No	29	94.3

**Health care-related data and Timing of first ANC attendance**: four hundred eighty-six (95.5%) of the respondents did not pay to get ANC services. The majority (92.7%) and 250 (49.1%) of the respondents said that the ANC service provider respected them and could afford transportation fees, respectively. Among 509 participants, 337 (66%) begun their first ANC visit after 16 weeks of gestation. The mean (±SD) gestational age was 19.9 (±6.1SD) weeks, ranging from 8^th^ weeks to 38^th^ weeks of gestational age at the first ANC booking.

**Factors associated with late ANC initiation**: after adjusting age, maternal education, gravidity, pregnancy intention, knowledge of timely initiation and got advised to have ANC as confounding factors, mothers aged 25 and above years, attended below secondary school education, unplanned pregnancy, pregnancy recognition by missing period, perceived the right time of ANC after 4 months, and did not get advice to have ANC were significantly associated with Late ANC initiation in the multivariable logistic regression analysis (Table 4).

**Table 4:** factors associated with late first ANC initiation among pregnant women at selected public health institutions in southwest Ethiopia

Variables	Categories	ANC Initiation		COR (95%CI)	AOR (95%CI)
		Late	Early		
**Age**	< 25 years	116	84	1	1
	≥ 25 years	221	88	1.82(1.25-2.64)	1.58(1.02-2.48) **
**Maternal education**	No education	158	94	0.93(0.54-1.60)	0.99(0.56-1.78)
	Primary (1-8)	85	37	1.27(0.69-2.35)	1.30(0.67-2.50)
	Secondary (9-12)	47	15	1.73(0.82-3.68)	2.33(1.05-5.19) **
	College and above	47	26	1	1
**Marital status**	Married	314	165	1	1
	Unmarried	23	7	1.73(0.73-4.11)	1.58(0.63-3.96)
**Household wealth index**	Poor	71	28	1.26(0.84-1.89)	1.26(0.82-1.96)
	Medium	66	27	1.46(0.86-2.48)	1.49(0.85-2.62)
	Rich	71	38	1	1
**Gravidity**	Primigravida	102	67	1	1
	Multigravida	235	105	1.47 (1.01-2.16)	1.16(0.73-1.84)
**Pregnancy intention**	Planned	232	148	1	1
	Unplanned	105	24	2.79(1.71-4.55)	2.25(1.34-3.77) *
**Pregnancy recognition**	Missing period	206	124	0.61(0.41-0.90)	0.61(0.39-0.93)**
	Urine test	131	48	1	1
**Perceived right time to start ANC**.	Within 4 months	224	143	1	1
	After 4 months	113	29	2.49(1.57-3.93)	2.29(1.36-3.85) *
**Knowledge of timely initiation**.	Good	152	104	1	1
	Poor	185	68	1.86(1.28-2.70)	1.25(0.81-1.93)
**Got advised to have ANC**	Yes	144	100	1	1
	No	193	72	1.86(1.28-2.70)	1.64(1.10-2.45) **

CI: Confidence Interval, COR: Crude odds ratio, AOR: Adjusted odds ratio, *p-value < 0.05, **p-value < 0.01

## Discussion

This study aimed to assess the magnitude and contributors of late antenatal care initiation among pregnant women at selected public health institutions of the Bench-Sheko Zone in southwest Ethiopia. As a result, 66%, 95% CI (62.7%, 70.3%) were initiated their ANC lately. This study was in line with 61.1% in Wolayta, 67.3% in Mekele, and 64.5% in Gondar [9, 14, 25] studies in Ethiopia. It was higher than the 42% in Addis Ababa [9], and 48.6% in Bahir Dar [18] studies in Ethiopia, and 56.2% in Myanmar [20]. This might be due to socio-demographic, economic, and cultural differences, as evidenced by the fact that the majority of pregnant women had no formal education and more than half of the women living in rural areas were housewives as compared to Addis Ababa and northern Ethiopian residents. But it was lower than 72.8% in Halaba and 81.5% in Wollega [4, 15] studies in Ethiopia.

The mean gestational age of respondents in the current study was comparable with a previous study done in Arbaminch town, Ethiopia [26]. But, the finding was higher than the studies conducted at Gonder and Dilla towns in Ethiopia [25, 27]. Women aged 25 years and above were 1.6 times more likely to be late for the first ANC initiation compared with the age of < 25 years. This finding was supported by studies done in Halaba, Kembata Tembaro, and Gonder [4, 13, 27] in Ethiopia. The possible reason for older women delaying their first ANC might be due to low educational status, having poor knowledge of ANC, have experienced pregnancies without complications previously, and are less fearful, unlike younger women. The finding observed between pregnant mothers may have a right or wrong perception of the time of first ANC initiation. Respondents who perceived the right time after four months of gestational age were 2 times more likely to book late than those who perceived the right time before four months of pregnancy. This finding was consistent with previous reports from the Adigrat zone in Ethiopia, and Nigeria, which also reported a strong association between the perceived ANC schedule and the timing of the first ANC visit [14, 20]. A study from Addis Ababa also reported that women who perceived the right time after four months of gestational age were 6.6 times more likely to book late than those who perceived the right time before four months of pregnancy [9]. Another study conducted by Gudayu *et al*. revealed that the perception of respondents concerning the correct time of early initiation of ANC was highly associated with early initiation of ANC at the recommended time [27]. It could be that pregnant women usually practice what they perceive.

Women whose pregnancy was unplanned were 2 times more likely to book late for their first ANC visit as compared to those who had planned pregnancy. This finding was consistent with the study done in Myanmar, which stated that unintended pregnancy was more likely too late to use antenatal care services [21]. Furthermore, studies done in Addis Ababa, Kembata Tembaro, Adigerat, Arbaminch, and Dilla areas in Ethiopia indicated that unwanted pregnancies are more likely associated with late first antenatal care booking compared to pregnancy reported as wanted [9, 13, 14, 25, 26]. This could be due to pregnant women with an unplanned pregnancy that may probably have less interest in pregnancy and to get information about ANC from significant others. The respondent´s education was a predictor of the time of the ANC booking. Women who attended below secondary school were 2 times as likely to come late were compared to those who attended above secondary education. This finding was consistent with studies conducted in Kembata Tembaro and Arbaminch town in Ethiopia revealed that women´s low level of educational status was associated with late entry into ANC [13, 26]. Having a better awareness may enable women to seek ANC and utilize the service early in pregnancy [28]. Women who were not advised about the timing of the first ANC at the recommended time were 1.6 times more likely as compared to those who received advice on the recommended time of the first ANC booking. This finding was similar to studies conducted in Halaba and Dilla [4, 25] studies in Ethiopia. This showed that awareness of the time of ANC booking increases the timely initiation of ANC service by pregnant women.

Pregnant mothers who recognized their pregnancy by missing periods were 39% less likely to commence ANC late. This was slightly lower than the findings in Halaba and Gonder, which indicates that pregnant women who used urine tests as a means of pregnancy recognition were nearly five times more likely in Halaba and two times more likely in Gonder to come early when compared to those who used a missing period [4, 27]. This difference could be for the fact that urine is done in health institutions and mothers are initiated to start ANC at the time they come to confirm pregnancy. The majority (92.7%) of respondents had respectful care in their previous antenatal care follow-up. This finding was in line with 91.85% of respectful family planning services in the Sidama zone, southern Ethiopia [29]. But, it was much higher than 38.4% respectful maternity care in Harar hospitals, Eastern Ethiopia [30], and 35.8% respectful maternity care in West Shoa zone, Oromia region, Ethiopia [31]. The variation observed could be the sociocultural and the compassionate respectful care training provision levels across different areas in Ethiopia. The study populations of this study and a study done in the Sidama zone have almost similar sociocultural characteristics; that is why the proportions obtained in the two studies become nearly similar. Since both studies are done in the southern region of Ethiopia. The study populations of Harar hospitals and the West Shoa zone have almost similar sociocultural characteristics; that is why the proportions obtained in the two studies become nearly similar.

**Limitations**: first, the cross-sectional nature of the study; does not allow us to ascertain causality. Second, the study only considers pregnant women attending public health institutions. As a result, a significant number of pregnant who were attended private health institutions were overlooked.

## Conclusion

The majority of pregnant women initiate their first antenatal care lately. We can conclude that late antenatal care initiation is a major problem in the study area. Therefore, providing continuous health education on the importance of initiating antenatal care visits early to prevent unwanted pregnancy outcomes is an important segment of intervention that can be done through health extension workers.

### What is known about this topic

- Antenatal care from skilled providers at health facilities is a priority in ensuring and enriching maternofetal health;
- Early commencement of antenatal care by pregnant women as well as regular visits has the potential to affect maternal and fetal outcomes positively. However, late initiation of ANC may lead to undetected or late detection of fetal and maternal health problems;
- Despite antenatal care service is a less resource-intensive intervention provided throughout the pregnancy period to reduce maternal mortality, however, most pregnant women in sub-Saharan Africa have begun ANC service utilization lately.

### What this study adds

- This study revealed that the majority (66%) of pregnant women initiate their first antenatal care lately;
- The study also identified, the increased age of mothers, unplanned pregnancy, perceived right time of ANC after 4 months, attended below the secondary school, and did not get advice to have ANC were the factors associated with late ANC initiation.
